# Violence as a Barrier for HIV Prevention among Female Sex Workers in Argentina

**DOI:** 10.1371/journal.pone.0054147

**Published:** 2013-01-16

**Authors:** María A. Pando, Romina S. Coloccini, Elena Reynaga, Marcelo Rodriguez Fermepin, Lucia Gallo Vaulet, Tadeusz J. Kochel, Silvia M. Montano, María M. Avila

**Affiliations:** 1 Instituto de Investigaciones Biomédicas en Retrovirus y SIDA (INBIRS), Facultad de Medicina, Universidad de Buenos Aires, Buenos Aires, Argentina; 2 Consejo Nacional de Investigaciones Científicas y Técnicas (CONICET), Buenos Aires, Argentina; 3 AMMAR, Asociación de Mujeres Meretrices de Argentina, Buenos Aires, Argentina; 4 Inmunología Clínica, Departamento de Bioquímica Clínica, Facultad de Farmacia y Bioquímica, Universidad de Buenos Aires, Buenos Aires, Argentina; 5 U.S. Naval Medical Research Unit No. 6, Lima, Peru; University College London Institute of Child Health, United Kingdom

## Abstract

**Background:**

Violence against female sex workers (FSWs) has been increasingly reported as an important determinant of HIV infection risk. This study explores the frequency of different violent experiences (sexual abuse, rejection, beating and imprisonment) among FSWs in Argentina and its association with condom use and HIV and *T. pallidum* prevalence.

**Methods:**

A convenience sample of 1255 FSWs was included in a cross-sectional study conducted between October 2006 and November 2009.

**Results:**

Sexual abuse was reported by 24.1% (219/907) of women. A total of 34.7% (42/1234) reported rejection experiences, 21.9% (267/1215) reported having been beaten and 45.4% (561/1236) stated having been arrested because of their sex work activity. There was a higher frequency of inconsistent condom use with clients among FSWs who had experienced sexual abuse, rejection, and police detention. A higher frequency of HIV and *T. pallidum* infection was detected among FSWs who reported having been arrested by the police.

**Conclusion:**

The study shows for the first time the frequency of different violent situations among FSWs in Argentina. The association between violence against sex workers, condom use and STI prevalence demonstrated here calls for measures to reduce stigma and violence against FSWs. Such violent experiences may increase vulnerability to STI through coerced unprotected sex.

## Introduction

Although sex work is a universal phenomenon, it is also illegal in most countries. Consequently, sex workers in many regions may have little or no legal protection and may be susceptible to human rights violations, including different kinds of violence [1]–[3]. As defined by the World Health Organization (WHO), violence is “the intentional use of physical force or power”, including not only more obvious violent acts but also those that result from a power relationship, in addition to threats and intimidation [4]. Female sex workers (FSWs) are particularly vulnerable to different kinds of violence in line with the high prevalence of gender-based violence occurring in many countries [5]. Gender-based violence has generally been recognized as a key public health problem associated with high rates of morbidity and mortality as a result of its psychological and medical sequelae including sexually transmitted infections (STI) [6].

A high frequency of different violent experiences against FSWs has been increasingly reported in several settings, also as an important determinant of HIV infection risk [7]–[14]. Previous studies that FSWs involved in violent situations are forced to prioritize their security over attempts to negotiate condom use [6], [15]–[16]. This problem is compounded by current legal systems that impede HIV prevention campaigns in some regions. For example, the police force of some countries often uses condom possession by FSWs as evidence for prosecution [9], [15], [17]–[18].

In Argentina, sex work *per se* is not illegal as long as it is conducted privately. What is illegal is the exploitation of persons involved in sex work. However, even though sex work is not criminalized, it is prohibited in some cities according to local public space regulations. In addition, Argentinean police force often uses possession of condoms as evidence to justify the arrest of individuals. This creates an environment where reporting sexual and physical violence (perpetuated by clients, police, or family members) may be very difficult (E. Reynaga, AMMAR, personal communication, 2006).

Argentina has a concentrated epidemic with HIV prevalence higher than 5% in more than one defined subpopulation (e.g. transgender sex workers, 33.9% (38/112); men who have sex with men, 10.3% (156/1518)) and lower than 1% in pregnant women [19]–[20]. A previous cross-sectional study on Argentinean FSWs between 2000 and 2002 showed an HIV prevalence of 3.2% (20/625) [21]. This study demonstrated that approximately 18% (113/625) of FSWs did not use condoms regularly with clients and that 87% (294/336) were engaged in unprotected sex with non-commercial partners. These high rates of inconsistent condom use demands further, in-depth studies of individual, interpersonal and environmental factors that may influence condom negotiations specifically.

Personal communications with Argentinean FSWs revealed that different kinds of violent situations are key concerns for them (E. Reynaga, AMMAR, personal communication, 2006). However, lack of data was observed regarding the frequency of such events as well as its impact on condom negotiation and on HIV and other STI rates in Argentinean FSWs. A new cross-sectional study aimed to update the prevalence of HIV and *T. pallidum* among Argentinean FSWs during 2006–2009 [20]. As part of this study, the frequency of different kinds of violence experienced by FSWs was explored. The aim of the present study was to estimate the frequency and the effect of different kinds of violent situations on condom negotiation, and HIV and *T. pallidum* prevalence rates.

## Methods

### Setting & Study Design

This cross-sectional, observational study was conducted among FSWs in the Argentinean cities of Buenos Aires, Santiago del Estero, Viedma, Rosario, Paraná, Córdoba, La Plata and Mendoza between October 2006 and November 2009 [20]. The project was coordinated by the INBIRS in collaboration with the Argentinean Female Sex Workers Non-Governmental Organization (*AMMAR*). In each city FSWs were contacted by trained peers from AMMAR in the street, bathhouses, nightclubs and brothels and offered to participate in the study consisting of the diagnosis of HIV and *Treponema pallidum* counseling and a structured questionnaire. Those who accepted were referred to the recruitment places specially equipped for the enrolment. These FSWs were included in the study if they met the participantś inclusion criteria. To avoid duplicating data, each participant was enrolled using a code with a letter indicating gender (F, female or M, Male), the two first letters of the first name, the two first letters of the surname, and the birth date (dd/mm/yyyy, e.g., a FSW named Maria Perez, born on February 1^st^ 1976, the code was FMAPE 01021976). *Ethics Statement.*

This research study was approved by the Institutional Review Board and the Scientific Ethics Committee of the School of Medicine, University of Buenos Aires and NAMRU-6 (NMRCD.2007.0021, Epidemiology of HIV in Latin American Countries) and conducted in compliance with all federal regulations governing the protection of human subjects. All potential participants underwent an informed consent procedure prior to entering the study and provided written consent.

### Enrolment Criteria, Procedures and Variables

Women older than 18 years of age who reported having exchanged sex for money during the previous six months were considered eligible for the survey. Volunteers who provided a written informed consent were invited to receive HIV and *T. pallidum* testing, pre-test counseling and were interviewed using a structured questionnaire. The interview took approximately 20 minutes. The questionnaire was managed by a peer previously trained and included several socio-demographic characteristics like age, nationality, formal education level, civil status and place of living (homeless or living in tenements, house, or apartment). Other set of variables searched for the presence of non-commercial sex partners, condom use with these partners (defining regular as “always use condom” and irregular as “frequently, sometimes or never use condom”), and number of children. Other sections of the questionnaire assessed issues related to sex work activity: place of clients contact, years in sex work activity, number of clients per week and condom use with clients. Following the definition of violence provided by WHO (including physical violence, threats and intimidation), different questions addressed situations related with violence. FSWs were asked if they had agreed to have their first sexual intercourse or had been sexually abused and if they had had other events of sexual abuse in their lives. In both questions sexual abuse was explained as a non-consented sexual relationship that includes penetration (oral, anal or vaginal). In case of abuse, women reported who perpetuated it. Participants also reported if they had been rejected and/or beaten as a result of their sex work activity. In both cases, participants reported who perpetuated the rejection and/or the beating. Finally, FSWs were asked about imprisonment events as a consequence of their sex work activity.

### HIV and *T. Pallidum* Diagnostic Procedures

Blood samples were collected at each site and referred to the INBIRS for processing. HIV diagnosis was assessed at the INBIRS using two screening tests (GENSCREEN Ultra HIV Ag-Ab, Bio-Rad, Marnes-la-Coquette, France; SFD HIV 1/2 PA, Bio-Rad FUJIREBIO INC, Tokyo, Japan) and reactive samples were further confirmed by Western blot assay for HIV-1 (New Lav Blot I, Bio-Rad, Marnes-la-Coquette, France). *T. pallidum* infection was determined at *“Hospital de Clínicas José de San Martin*” by non-treponemal and treponemal assays (VDRL, Wiener Laboratorios, SAIC, Rosario, Argentina; TPHA, Biokit SA, Barcelona, Spain). An indirect immunofluorescence test (FTA-abs, Inmunofluor Biocientifica SA, Argentina) was performed to clarify discordant results.

The results of HIV and *T. pallidum* diagnoses were sent to each recruitment site and delivered to the volunteers in a confidential post-test counseling session. All HIV and/or *T. pallidum* positive individuals were referred to local hospitals for medical care and treatment.

### Data Analysis

Questionnaires from different recruitment sites were merged into a database. Data analysis was performed using SPSS (SPSS for Windows 2006, Version 15). Baseline characteristics were described using mean or medians and standard deviation or interquartile ranges (IQRs) for continuous variables and counts and percentages for categorical data. Chi-square test or Fisher’s exact test were used to compare proportions. All reported p values were two-sided; p values <0.05 were considered to be statistically significant. Due to missing data, tables present total numbers for each item.

The association of different violent experiences with condom use (separately with clients and non-commercial partners), HIV and *T. pallidum* infections was initially studied through univariate analysis that generated crude odds ratio (OR) and 95% confidence intervals (CIs). Major demographic characteristics (age, nationality and formal education level) as well as HIV status and place of work were included in a stepwise regression logistic model to allow the estimation of adjusted OR. Age was treated as a continuous variable in the model.

## Results

### Socio-demographic, Sex Work Characteristics and HIV/STI Prevalence in the Study Population

A total of 1255 FSWs from different cities in Argentina participated in the survey. We previously reported the socio-demographic characteristics of the study population [22]. Briefly, even when the majority of the FSWs were Argentinean (71.1%), a high frequency of foreigners was found, in particular Paraguayan (262/1252, 20.9%). Mean age was 33.5 years old (SD 11.1, range 18–74). In relation to formal education level, approximately 10% of the FSWs did not have any formal education, 34.4% attended high school, and 4.6% had completed higher education. Most of the women were single (60.4%); however, 50% stated having a sexual partner. Regular (always) use of condom with sexual partners was reported by 17.6% of women. A total of 86% of the FSWs reported to have children (median number of children: 3; IQRs: 2–4; range: 1–15). A total of 24.1% (298/1239) of women lived in poverty (defined as homeless or living in tenements, slums or shanty towns).

Regarding their sex work activity, FSWs had a median number of 4 years in the sex work activity (IQRs: 1–10) and 12 clients per week (IQRs: 6–20). The majority of participants contacted the clients on the streets (53.9%) and 31.8% contacted them in private places like their own residence or houses, specially conditioned for the activity. The regular use of condom with clients was reported by 88.6% of the FSWs, being significantly higher than its use with non-commercial sexual partners (88.6% vs. 17.6%, p<0.0001) (Table 1).

**Table 1:** Socio-demographics characteristics of 1255 FSWs recruited in nine cities of Argentina between 2006 and 2009.

		n/N	Frequency (%)
Nationality	Argentinean	890/1252	71.1
	Other	362/1252	28.9
Formal Education Level	Any	121/1249	9.7
	Elementary school	698/1249	55.9
	High school	372/1249	29.8
	More than high school	58/1249	4.6
Civil status	Single	756/1251	60.4
	Divorced	174/1251	13.9
	Living with a partner	146/1251	11.6
	Married	128/1251	10.2
	Widow	47/1251	3.8
Have a sexual partner	Yes	624/1249	50.0
Regular use of condom with sexual partner	Yes	110/624	17.6
Have children	Yes	1053/1224	86.0
Place of clients contact	Streets	676/1255	53.9
	Private places	399/1255	31.8
	Bar	180/1255	14.3
Regular use of condom with clients	Yes	1100/1241	88.6

The overall prevalence of HIV infection was 2% (25/1255, 95%CI 1.2–2.8) and of *T. pallidum* infection 22.4% (245/1094, 95%CI 19.9–24.9) [22].

### Experience of Different Kinds of Violence Among FSWs: Univariate Analysis

Rates of different violent experiences are shown in Table 2. Sexual abuse was reported by a total of 24.1% of the women, 7.3% experienced sexual abuse during their first ever sexual encounter, 20.0% were sexually abused at other times in their lives, and 3.4% of the women reported both. With regards to those FSWs who had been sexually abused during their first ever sexual encounter, family members were identified as the perpetrators in the majority of cases (59.0%). Less frequently, unknown individuals (21.3%) or acquaintances (18%) were also identified. Among those women who reported sexual abuse at other times in their lives, the majority of participants also identified relatives as perpetrators (42.6%). Less frequently acquaintances (20.1%), clients (18.3%) or unknown individuals (15.4%) were identified as perpetrators. The age of first ever sexual encounter was significantly lower in those who were abused when compared with those who consented having sex (mean 13.3 vs. 15.8, respectively, p<0.001).

**Table 2:** Frequency of rejection and violent experiences in FSWs recruited in nine cities of Argentina between 2006 and 2009.

	n/N	%
Sexual abuse		
During first sexual experience	68/932	7.3
At another stage in life	182/910	20.0
During first sexual experience or at another stage in life	219/907	24.1
During first sexual experience and at another stage in life	31/901	3.4
Experienced rejection because of sex work	428/1234	34.7
History of being beaten because of sex work	267/1215	22.0
History of being arrested because of sex work	561/1236	45.4
Number of violent episodes experienced*		
0	263/870	30.2
1	277/870	31.8
2	179/870	20.6
3	95/870	10.9
4	56/870	6.4

* 1 point awarded for each type of episode experienced (sexual abuse, being beaten, feelings of rejection, being arrested due to sex work activity).

A total of 34.7% of the women reported having been rejected because of their sex work, mainly by unknown individuals (49.1%), but also by the police (27.8%), neighbors (27.6%), relatives (27.2%) and partners (20.1%). In addition, 22.0% of the women reported having been beaten because of their sex work mainly by unknown individuals (46.8%), but also by partners (30.4%), clients (28.8%) and the police (23.6%). Nearly half of the women (45.4%) reported having been arrested because of their sex work activity.

Considering all the situations of violence, approximately 70% of the participants had experienced at least one of them (sexual abuse, rejection, being beaten or arrested) in their lives.

### Violence and Condom Use: Bivariate and Multivariate Analyses

Bivariate and multivariate analyses of the association between condom use and history of violent experiences are described in Table 3. Bivariate analyses revealed that condom use was highly irregular among those FSWs who had experienced some kind of violent situation, being mostly statistically significant in relation to the use of condom with clients. In the multivariate model (adjusted by nationality, HIV status, formal education level, age and place of work), a history of sexual abuse (during their first sexual intercourse, during their lives or both) and being rejected remained significantly associated with inconsistent use of condom with clients. In relation with non-commercial partners, significantly high irregular condom use was observed only among those who reported sexual abuse in their lives. Additionally, there was a significant positive trend between the number of different violent experiences (0–4) and the frequency of inconsistent condom use with clients.

**Table 3:** Bivariate and multivariate analysis of association between violent experiences and condom use in FSWs recruited in nine cities of Argentina between 2006 and 2009.

		Irregular use of condoms					
		With non-commercial partner			With clients		
		Frequency (%)	OR (95%CI)	aOR (95%CI)	Frequency (%)	OR (95%CI)	aOR (95%CI)
Sex abuse during the first sexual experience	Yes	88.6	1.6 (0.5–4.7)	1.4 (0.7–2.6)	**24.2**	**2.5 (1.4–4.5)**	**1.5 (1.1–2.0)**
	No	82.9			**11.5**		
Sex abuse during lifetime	Yes	**89.7**	**2.1 (1.1–4.1)**	**1.4 (1.0–1.9)**	**20.8**	**2.4 (1.6–3.7)**	**1.5 (1.2–1.8)**
	No	**80.6**			**9.8**		
Sex abuse during the first sexual experience OR during lifetime	Yes	88.1	1.7 (0.9–3.2)	1.3 (0.9–1.8)	**20.5**	**2.4 (1.6–3.6)**	**1.5 (1.2–1.8)**
	No	80.9			**9.7**		
Have you been rejected because of your sex work activity?	Yes	85.3	1.3 (0.9–2.1)	1.2 (0.9–1.5)	**16.4**	**2.1 (1.4–2.9)**	**1.4 (1.1–1.6)**
	No	81.1			**8.7**		
Have you been beaten because of your sex work activity?	Yes	85.8	1.4 (0.8–2.3)	1.1 (0.8–1.5)	14.2	1.5 (1.0–2.2)	1.1 (0.9–1.3)
	No	81.5			10.2		
Have you been arrested because of your sex work activity?	Yes	84.4	1.3 (0.9–2.0)	1.0 (0.8–1.3)	**14.7**	**1.9 (1.3–2.7)**	1.1 (0.9–1.4)
	No	80.7			**8.5**		
Number of violent episodes experienced*	**0**	**77.7**	1	1	**8.4**	1	1
	**1**	**83.2**	1.1 (0.9–1.2)	0.9 (0.5–1.5)	**10.1**	1.2 (0.7–2.0)	0.7 (0.5–1.1)
	**2**	**85.1**	1.1 (1.0–1.2)	0.9 (0.5–1.6)	**13.6**	1.6 (0.9–2.8)	0.9 (0.6–1.4)
	**3**	**85.7**	1.1 (0.9–1.3)	0.9 (0.4–1.8)	**14.7**	1.7 (0.9–3.3)	1.1 (0.6–1.8)
	**4**	**94.1**	**1.2 (1.1–1.4)**	2.1 (0.6–6.9)	**26.8**	**3.2 (1.8–5.7)**	**1.9 (1.1–3.3)**

aOR: adjusted by nationality (Argentinean vs. other), HIV status, formal education level (primary or less vs. high school or more), age and place of work (street vs. other).

### Violence and HIV/STI Prevalence: Bivariate and Multivariate Analyses

Bivariate and multivariate analyses of the association between HIV/*T. pallidum* prevalence and a history of violent experiences are shown in Table 4. A statistically significant association between HIV infection and history of rejection or being arrested was demonstrated by the bivariate analysis. This association remained significant after adjusting for nationality (Argentinean vs. other), formal education level (primary or less vs. high school or more), age and place of work (street vs. other). In addition, a significant association was detected between the number of different violent experiences and HIV prevalence but not remaining significant after adjustment (Table 3). A statistically significant higher frequency of *T.pallidum* infection was observed in those women who had been arrested because of their sex work activity. This association remained significant on the multivariate analysis using the same model as in HIV infection.

**Table 4:** Bivariate and multivariate analysis of violent experiences and HIV/*T. pallidum* infection in FSWs recruited in nine cities of Argentina between 2006 and 2009.

		HIV infection			*T. pallidum* infection		
		Frequency (%)	OR (95%CI)	aOR (95%CI)	Frequency (%)	OR (95%CI)	aOR (95%CI)
Sex abuse during the first sexual experience	Yes	0	–	–	29.1	1.7 (0.9–3.1)	1.2 (0.9–1.7)
	No	1.7			19.7		
Sex abuse during lifetime	Yes	3.3	2.4 (0.9–6.6)	1.5 (0.9–2.5)	25.2	1.4 (0.9–2.1)	1.1 (0.9–1.4)
	No	1.4			19.7		
Sex abuse during the first sexual experience OR during lifetime	Yes	2.7	2.1 (0.8–5.8)	1.4 (0.8–2.4)	24.3	1.3 (0.9–1.9)	1.1 (0.9–1.3)
	No	1.3			19.8		
Have you been rejected because of your sex work activity?	Yes	**3.5**	**3.6 (1.6–8.4)**	**1.8 (1.1–2.7)**	23.1	1.1 (0.8–1.4)	1.0 (0.9–1.2)
	No	**1.0**			21.8		
Have you been beaten because of your sex work activity?	Yes	3.0	1.9 (0.8–4.5)	1.2 (0.8–1.9)	25.1	1.2 (0.8–1.7)	1.0 (0.9–1.2)
	No	1.6			21.9		
Have you been arrested because of your sex work activity?	Yes	**3.2**	**4.4 (1.6–12.0)**	**1.8 (1.1–3.0)**	**29.5**	**2.1 (1.6–2.8)**	**1.5 (1.2–1.7)**
	No	**0.7**			**16.5**		
Number of violent experiences	**0**	**0.4**	1	1	17.3	1	1
	**1**	**1.4**	3.8 (0.4–33.8)	0.8 (0.3–2.3)	22.7	1.3 (0.9–1.9)	1.1 (0.8–1.5)
	**2**	**2.2**	5.9 (0.7–52.2)	1.3 (0.5–3.4)	20.9	1.2 (0.8–1.8)	0.9 (0.6–1.4)
	**3**	**2.1**	5.5 (0.5–60.4)	1.2 (0.3–4.2)	21.5	1.2 (0.7–2.1)	1.1 (0.7–1.8)
	**4**	**5.4**	**14.1 (1.5–133.0)**	2.9 (0.9–9.2)	29.2	1.7 (1.0–2.9)	1.1 (0.6–2.0)

aOR: adjusted by nationality (Argentinean vs. other), formal education level (primary or less vs. high school or more), age and place of work (street vs. other).

## Discussion

Our results demonstrate, for the first time in Argentina, an alarming prevalence of violent events reported by women involved in sex work, with 70% of participants declaring having experienced at least one form of violence. Furthermore, FSWs who reported having experienced certain forms of violence were less likely to use condoms with clients. Moreover, participants who had experienced rejection were more likely to be HIV positive, and those who had been arrested were more likely to be positive for HIV or *T. pallidum* infections.

Previous studies among FSWs have estimated that around 40–70% of them suffer physical or sexual violence [6]–[14], [23]. Although sex work is widespread in Latin America, only few studies have explored this issue in the region. One study from Nicaragua reported high frequency of physical or sexual violence among FSWs (45.5%) [24] but another study conducted in Brazil suggested that having been physically forced to have sexual intercourse increases the possibility of being infected by HIV [25]. Only one previous study estimated the frequency of violence against women attending a hospital in Buenos Aires, Argentina. This showed that 45% of the participating women had suffered gender-based violence, including psychological, physical or sexual violence [26]. Comparing this data with our study is difficult since both studies used different parameters to measure events of violence.

Four categories of violence among women were explored here: sexual abuse, perception of rejection, being beaten and being arrested by the police. Of those participants reporting sexual abuse (24%), the high percentage of women who had been abused by members of the family, relatives or acquaintances, either during their first sexual intercourse or at any stage in their lives is particularly alarming. These results, together with the fact that approximately 7% of women reported having been involved in sex work activity because of family coercion (data not shown), suggest that their families and/or childhood environment of FSWs may be an important determinant of risk behavior during subsequent sex work activity. However, the association of sexual abuse and sexual risk behavior may not be unique to the sex worker population. Previous studies on women not involved in sex work activities have observed that a history of sexual abuse during childhood was associated with a significantly lower likelihood of condom use and a higher risk of contracting HIV and other STIs [27]. These results suggest that reticence in condom negotiation is not a feature of sex work activity but it is strongly associated with previous sexual abuse. It is also important to consider that approximately 18% of the women recruited in this study reported sexual abuse perpetuated by clients. These situations make women vulnerable to condom negotiation not only in the particular relationship where women are abused, but also for their future. In fact, higher frequencies of irregular use of condoms with clients were found among those women who reported sexual abuse. Further qualitative research will be essential and valuable to clarify the relationship between sexual abuse and condom negotiation among FSWs, and in guiding future interventions among this vulnerable group.

In relation to the reports on rejection due to sex work activity, it is important to consider that it is difficult to objectively measure rejection. Perception of rejection may differ among women, either by under or over-estimation and it may differ according to cultural backgrounds. Nevertheless, 35% of these participants reported perceiving rejection, highlighting that it is an important issue among this group. Previous personal communications with Argentinean FSWs support this observation since such rejection by unknown individuals tends to occur mostly in the street and consists of insults or comments with negative connotations (E. Reynaga, AMMAR, personal communication, 2011). These results suggest that these FSWs are prone to marginalization. While not measured here, sex workers revealed that such marginalization could be observed to be perpetrated by some health-care system staff, as previously reported by other studies [28]. Determining the effect of such marginalization on the access to health-care and other services requires further studies, which is a priority in this population. Regarding sexual abuse, FSWs who reported experiences of rejection due to sex work were less likely to use condoms with clients. Future HIV/STI preventive measures could perhaps target this determinant of condom negotiation.

A high frequency of women (22%) reported having been beaten because of their sex work activity. Although they were mostly beaten by unknown individuals, a high percentage also reported partners as responsible for these violent incidents. Violence against women by intimate partners and its association with lower rates of condom use has been described previously [29]. Such physical violence may increase vulnerability to STI through coerced unprotected sex. Previous studies have observed that sex workers are forced to prioritize the immediate threat or fear of violence over attempts to insist on condom use. One study described how men who perpetrated intimate partner violence were significantly less likely to report using condoms with their female partners [30]. No published data from Argentina about physical violence or sexual abuse against FSWs, or women in general, and the influence on condom use is available for comparison with our study.

While sex work in Argentina is not illegal, ambiguities in the law exist due to public-space regulations and sex workers often have an uncertain relationship with law-enforcement bodies. Our results confirm this fact with nearly half of the study population reporting having been arrested because of their sex work activity. Most alarmingly, FSWs recruited in this study stated that policemen are responsible for rejecting and beating some of them. Studies from other countries revealed that violence against FSWs perpetrated by policemen include both the use of physical force and coerced sex in exchange for freedom [9]. Evidence from other studies also suggests that women who experienced violence perpetrated by the police may be less likely to access to police or judicial support [23]. Our data among women who were arrested because of their sex work showed higher frequencies of HIV and *T. pallidum* infections. Although condom use frequency was not statistically related with imprisonment after adjustment by demographic variables, lower rates of condom use, in particular with commercial partners, were detected among women who were arrested. These results suggest that this kind of violent situation can have an impact on condom negotiation, in particular with clients.

Some potential limitations should be considered in our study. Regarding external validity, as convenience sampling was used; this study may have sampling biases. Another factor to be considered is that no previous estimations of the number of FSWs in Argentina have ever been performed due to the often clandestine nature of sex-work. It is also important to consider that the recruitment period was long (three years).Though no period effect that could influence variations in recruitment or characteristics of the recruited FSWs were registered, we cannot be sure that some undetectable factors could bias the sampling. In addition internal validity of the study could be also affected. Given the use of self-reports to measure violent episodes, this study may be affected by a recall bias. Previous studies acknowledged that sex workers may underestimate situations of violence and fail to report the most common episodes of violence such as slapping, considering those events only as “bad dates” [6], [31]. In relation to condom use, the possibility of over-reporting condom use because of perceived stigmatization must be considered. Finally, even when a code that includes name and surname initials and date of birth was used, biometrics measures, such as fingerprints, should be used in order to avoid duplicating participants. Even with these limitations, this study provided very important information regarding violence among FSWs that can address future in-depth studies that the current research could not answer.

While, the cross-sectional design of this study did not allow us to establish causality, hypotheses can be suggested to account for the associations among violence, STI infection rates and STI risk behavior that have been observed here. Violence could enhance vulnerability to HIV and other STIs through different mechanisms. Examples include having unprotected intercourse during sexual abuse or being unable to negotiate condom use during a violent situation, or lack of motivation to use condoms after experiencing violence. Prospective follow-up of FSWs should be considered in the future in order to study causality.

The association between violence against sex workers, lower condom use and higher STI prevalence demonstrated here calls for measures to reduce violence against FSWs in Argentina. Previous studies have shown that reducing violence against FSWs can facilitate condom use and thus possibly reduce STI transmission among sex workers [17], [32]. Interventions to reduce violence against sex workers in Argentina could potentially include legal protection, removal of criminal sanctions on sex work activity, and education of the population, the police force, clients and other relevant subpopulations associated with FSWs. Further studies are essential to help implement, guide and assess the effectiveness of such interventions.

In summary, results from this study demonstrated, for the first time in Argentina, an alarming prevalence of violent situations among FSWs. Certain forms of violence were significantly associated with reduced condom use and higher HIV and *T. pallidum* prevalence. Our findings highlight the need for future HIV prevention efforts to incorporate interventions that target violence against women involved in sex work activities.

## References

[1] UNAIDS (2002) Sex work and HIV/AIDS. Geneva: Joint united Nations Programme on HIV/AIDS. Available: http://data.unaids.org/publications/IRC-pub02/jc705-sexwork-tu_en.pdf.

[2] Ahmad K (2001) Call for decriminalization of prostitution in Asia. Lancet 358: 643. 11530157 10.1016/S0140-6736(01)05819-6

[3] Rekart ML (2005) Sex-work harm reduction. Lancet 366: 2123–2134. 16360791 10.1016/S0140-6736(05)67732-X

[4] Krug EG, Dahlberg LL, Mercy JA, Zwi AB and Lozano R (2002). World report on violence and health. Geneva, World Health Organization. Available: http://whqlibdoc.who.int/publications/2002/9241545615_eng.pdf.

[5] Watts C, Zimmerman C (2002) Violence against women: global scope and magnitude. Lancet 359: 1232–1237. 11955557 10.1016/S0140-6736(02)08221-1

[6] Shannon K, Kerr T, Strathdee SA, Shoveller J, Montaner JS, et al (2009) Prevalence and structural correlates of gender based violence among a prospective cohort of female sex workers. BMJ 339: b2939. 19671935 10.1136/bmj.b2939 PMC2725271

[7] Farley M, Baralm I, Kiremire M, Sezgin U (1998) Prostitution in five countries: violence and post-traumatic stress disorder. Feminism & Psychology 8: 405–426.

[8] Rekart ML (2005) Sex-work harm reduction. The Lancet 366: 2123–2134. 10.1016/S0140-6736(05)67732-X 16360791

[9] World Health Organization (2005). Violence against women and HIV/AIDS: critical intersections. Violence against sex workers and HIV prevention. Information Bulletin Series, Number 3. Available: http://www.who.int/gender/documents/sexworkers.pdf.

[10] Swain SN, Saggurti N, Battala M, Verma RK, Jain AK (2011) Experience of violence and adverse reproductive health outcomes, HIV risks among mobile female sex workers in India. BMC Public Health 20 11: 357. 10.1186/1471-2458-11-357 PMC3116498 21599984

[11] Lang DL, Salazar LF, Diclemente RJ, Markosyan K (2012). Gender based violence as a risk factors for HIV-associated risk behaviors among female sex workers in Armenia. AIDS Behav jul 4 [Epub ahead of print]. 10.1007/s10461-012-0245-7 22760740

[12] Decker MR, Wirtz AL, Baral SD, Peryshkina A, Mogilnyi V, et al (2012) Injection drug use, sexual risk, violence and STI/HIV among Moscow female sex workers. Sex Transm Infect 88(4): 278–283. 22287530 10.1136/sextrans-2011-050171 PMC3628729

[13] Zhang C, Li X, Hong Y, Chen Y, Liu W, et al (2012) Partner violence and HIV risk among female sex workers in China. AIDS Behav 16(4): 1020–1030. 21598033 10.1007/s10461-011-9968-0 PMC6686181

[14] Ghimire L, Smith WC, van Teijlingen ER, Dahal R, Luitel NP (2011) Reasons for non-use of condoms and self-efficacy among female sex workers: a qualitative study in Nepal. BMC Womeńs Health 11: 42. 21943102 10.1186/1472-6874-11-42 PMC3206429

[15] Rhodes T, Simic M, Baros S, Platt L, Zikic B (2008) Police violence and sexual risk among female and transvestite sex workers in Serbia: qualitative study. BMJ 337(7669): 560–563. 10.1136/bmj.a811 PMC2492575 18667468

[16] Decker MR, McCauley HL, Phuengsamran D, Janyam S, Seage GR III, et al (2010) Violence victimization, sexual risk and sexually transmitted infection symptoms among female sex workers in Thailand. Sex Transm Infect 86(3): 236–240. 20444745 10.1136/sti.2009.037846 PMC3578319

[17] Beattie TS, Bhattacharjee P, Ramesh BM, Gurnani V, Anthony J, et al (2010) Violence against female sex workers in Karnataka state, south India: impact on health, and reductions in violence following an intervention program. BMC Public Health 10: 476. 20701791 10.1186/1471-2458-10-476 PMC2931467

[18] Simić M, Rhodes T (2009) Violence, dignity and HIV vulnerability: street sex work in Serbia. Sociol Health Illn 31: 1–16. 19144087 10.1111/j.1467-9566.2008.01112.x

[19] Ministerio de Salud, Presidencia de la Nación (2010) Boletín sobre el VIH/sida en la Argentina. Año XIII, número 27. Argentina.

[20] Pando MA, Gómez-Carrillo M, Vignoles M, Rubio AE, dos Ramos Farias MS, et al (2011) Incidence of HIV-1 infection, antiretroviral drug resistance and molecular characterization in newly diagnosed individuals in Argentina. A Global Fund Project. AIDS Res Hum Retroviruses 27: 17–23. 20860532 10.1089/aid.2010.0013 PMC3034098

[21] Pando MA, Berini C, Bibini M, Fernández M, Reynaga E, et al (2006) Prevalence of HIV and other sexually transmitted infections among female commercial sex workers in Argentina. Am J Trop Med Hyg 74: 233–238. 16474076

[22] Pando MA, Reynaga E, Coloccini RS, Rodríguez Fermepín M, Kochel T, et al (2011) Prevalencia de la infección por el VIH y de Treponema pallidum en mujeres trabajadoras sexuales de Argentina. Rev Panam Salud Publica 30: 303–308. 22124688

[23] Shannon K, Csete J (2010) Violence, condom negotiation, and HIV/STI risk among sex workers. JAMA 304: 573–574. 20682941 10.1001/jama.2010.1090

[24] Decker MR, Mack KP, Barrows JJ, Silverman JG (2009) Sex trafficking, violence victimization, and condom use among prostituted women in Nicaragua. Int J Gynaecol Obstet 107: 151–152. 19577234 10.1016/j.ijgo.2009.06.002 PMC3623281

[25] Damacena GN, Szwarcwald CL, Borges de Souza J PR, Dourado I (2011) Risk factors associated with HIV prevalence among female sex workers in 10 Brazilian Cities. J Acquir Immune Defic Syndr 57(sup 3): S144–152. 10.1097/QAI.0b013e31821e9bf6 21857310

[26] Pontecorvo C, Mejía R, Aleman M, Vidal A, Majdalani MP, et al (2004) Detection of domestic violence against women.Survey in a primary health care clinic. Medicina (B Aires) 64: 492–496. 15637825

[27] Schacht RL, George WH, Davis KC, Heiman JR, Norris J, et al (2010) Sexual abuse history, alcohol intoxication, and women’s sexual risk behavior. Arch Sex Behav 39: 898–906. 19728070 10.1007/s10508-009-9544-0 PMC3159520

[28] Chakrapani V, Newman PA, Shunmugam M, Kurian AK, Dubrow R (2009) Barriers to free antiretroviral treatment access for female sex workers in Chennai, India. AIDS Patient Care STDS 23: 973–80. 19821725 10.1089/apc.2009.0035 PMC2832653

[29] Swan H, O’Connell DJ (2012) The impact of intimate partner violence on women’s condom negotiation efficacy. J Interpers Violence 27(4): 775–792. 21987514 10.1177/0886260511423240 PMC4451787

[30] Frye V, Ompad D, Chan C, Koblin B, Galea S, et al (2011) Intimate Partner Violence Perpetration and Condom Use-Related Factors: Associations with Heterosexual Men’s Consistent Condom Use. AIDS Behav 15: 153–162. 20069447 10.1007/s10461-009-9659-2 PMC4423735

[31] World Health Organization (2007). The third milestones of a global campaign for violence prevention report. Geneva: WHO.

[32] Carlson CE, Chen J, Chang M, Batsukh A, Toivgoo A, Riedel M, Witte SS (2012) Reducing intimate and paying partner violence against women who exchange sex in Mongolia: results from a randomized clinical trial. J Interpers Violence 27(10): 1911–1931. 22366477 10.1177/0886260511431439 PMC4269222

